# Health Care Use Before Multiple Sclerosis Symptom Onset

**DOI:** 10.1001/jamanetworkopen.2025.24635

**Published:** 2025-08-01

**Authors:** Marta Ruiz-Algueró, Feng Zhu, Anibal Chertcoff, Yinshan Zhao, Ruth Ann Marrie, Helen Tremlett

**Affiliations:** 1 Faculty of Medicine (Neurology), University of British Columbia and The Djavad Mowafaghian Centre for Brain Health, Vancouver, Canada; 2 Department of Internal Medicine (Neurology), Max Rady College of Medicine, Rady Faculty of Health Sciences, University of Manitoba, Winnipeg, Canada; 3 Department of Medicine and Community Health and Epidemiology, Faculty of Medicine, Dalhousie University, Halifax, Nova Scotia, Canada; 4 Nova Scotia Health, Halifax, Canada

## Abstract

**Importance:**

Health care use increases before multiple sclerosis (MS) onset. However, most studies have focused on the 5 to 10 years preceding the first demyelinating disease code from administrative data. Few studies have examined patterns before clinically determined MS symptom onset from clinical records.

**Objective:**

To examine health care use 25 years before MS symptom onset in a clinical cohort from British Columbia, Canada.

**Design, Setting, and Participants:**

This matched cohort study accessed data prospectively collected from January 1991 to September 2018. All data were released mid-2024 for analysis. The study was conducted in British Columbia using publicly funded universal health insurance data. Patients with MS were identified from MS clinic records and matched with up to 5 individuals randomly selected without replacement from the general population by sex, birth year, socioeconomic status, and postal code of residency.

**Main Outcomes and Measures:**

Linked clinical and administrative data were used to compare physician visit rates 25 years before MS onset using adjusted negative binomial models and 15 years before MS onset by *International Classification of Diseases, Ninth Revision* (*ICD-9*) chapter and physician specialty.

**Results:**

A total of 2038 patients with MS (mean [SD] age at symptom onset, 37.9 [10.9] years; 1508 female [74.0%]) and 10 182 matched individuals were included. All-cause physician visit rate ratios (RRs) for patients with MS were consistently elevated from 14 years before onset (adjusted RR [ARR], 1.19; 95% CI, 1.07-1.33), peaking the year before MS onset (ARR, 1.28; 95% CI, 1.21-1.35). The RRs for ill-defined symptoms and signs were consistently elevated 15 years before onset, exceeding 1.15 and peaking at 1.47 (95% CI, 1.36-1.58) the year before MS onset. Mental health–related RRs from 14 years before onset were significant (excluding years 7, 5, and 4), with RRs in the 3 years before MS onset ranging from 1.30 (95% CI, 1.05-1.58) to 1.38 (95% CI, 1.12-1.68). Sensory, musculoskeletal, and nervous system RRs were elevated 8, 5, and 4 years before onset, respectively, with, for example, a peak of 2.42 (95% CI, 1.90-3.07) for nervous system concerns the year before MS onset. By physician specialty, general practice visit RRs were significantly elevated in each of the 15 years before MS onset, reaching 1.23 (95% CI, 1.17-1.30) in the year before onset. Psychiatry visit RRs were elevated 12 years before onset (2.59; 95% CI, 1.23-5.47). Neurology and ophthalmology RRs were significantly higher up to 8 to 9 years before onset, peaking the year before MS onset at 5.46 (95% CI, 4.30-6.93) for neurology and 1.64 (95% CI, 1.30-2.08) for ophthalmology.

**Conclusions and Relevance:**

In this matched cohort study of people with and without MS, health care use was higher among patients with MS 14 to 15 years before MS symptom onset, suggesting that MS may have started earlier than previously thought. Mental health and psychiatric issues along with ill-defined signs and symptoms might be among the earliest features of the prodromal period preceding nervous system–related and neurologic visits by 7 to 11 years.

## Key Points

**Question:**

What are the patterns of health care use in the 25 years preceding the clinically determined onset of multiple sclerosis (MS)?

**Findings:**

In this cohort study of 2038 patients with MS and a matched cohort of 10 182 individuals without MS, all-cause physician visits were elevated 14 years before MS onset. Mental health–related visits and visits for ill-defined symptoms and signs were elevated 14 to 15 years before MS onset, followed by neurology and ophthalmology consultations (8-9 years before onset) and musculoskeletal, sensory, and nervous system visits (4-8 years before onset).

**Meaning:**

These findings suggest that MS may begin much earlier than previously recognized, with mental health–related issues as early indicators, highlighting opportunities for earlier identification and intervention.

## Teaser

This cohort study examines patterns in health care use 25 years before multiple sclerosis symptom onset in patients from British Columbia, Canada.

## Introduction

Prodromal phases are recognized for several chronic neurologic disorders, providing an opportunity for enhanced early detection and intervention strategies.^1,2,3,4^ While less is known about the potential prodromal phase in multiple sclerosis (MS), studies have shown that the years preceding the classical onset of MS are associated with higher health care use.^5,6,7,8,9,10,11,12,13,14,15^ In the 5 to 10 years before the onset of MS, people with MS have been shown to access the health system more frequently for headache, fatigue, sleep disorders, pain, gastrointestinal, and neuropsychiatric concerns.^5,9,10,12,16^ However, most studies examined the period before the first demyelinating event, as identified using administrative data, and few examined health care patterns before the clinically determined MS symptom onset date.^8^ In addition, few studies examined a period longer than 5 to 10 years before MS onset, such that the duration of prodromal symptoms remains unclear.^17^

Understanding the duration of the early phase of MS is crucial for identifying disease markers, enabling prompt recognition, and potentially informing future preventive strategies. Therefore, we accessed linked clinical and administrative data to investigate health care use patterns up to 25 years preceding the clinically determined MS symptom onset date.

## Methods

This cohort study gained access to health administrative and prescription dispensation data through Population Data BC. The University of British Columbia Clinical Research Ethics Board approved the study, including waiving consent for the use of administrative data. This report follows the Strengthening the Reporting of Observational Studies in Epidemiology (STROBE) reporting guideline.

We used data on physician and hospital visits from the Medical Services Plan Payment and the Discharge Abstract Database. Diagnoses were coded using *International Classification of Diseases, Ninth Revision* (*ICD-9*) and *International Statistical Classification of Diseases, Tenth Revision* codes, with additional British Columbia Diagnosis Codes and physician specialty available for physician visits. The dataset also encompassed demographic details such as sex, date of birth, region (urban or rural), and socioeconomic status based on neighborhood-level income linked to postal codes.^18^ Race and ethnicity data were not collected for this study. We also used each person’s registration dates in British Columbia’s health care system to confirm provincial residency. Data obtained from the British Columbia MS clinical database included the date of MS symptom onset. This date is recorded retrospectively by the neurologist after a medical history (and neurologic examination) is taken in the MS clinic. PharmaNet provided records of disease-modifying drug prescriptions filled at outpatient and community pharmacies, including dispensation dates and drug identification numbers. Data were available from January 1991 to September 2018, except for the disease-modifying drug prescriptions (available from January 1996, the first full year the disease-modifying drugs were approved for use in Canada). Population Data BC linked the clinical and administrative datasets using unique personal health numbers and released deidentified data to researchers in mid-2024.

### Cohort Selection

First, we identified persons with MS with a known date of MS symptom onset who were diagnosed using the prevailing international criteria at 1 of 4 participating MS clinics (eFigure 1 in Supplement 1).^19^ To examine the period before MS onset, the neurologist-determined date of MS symptom onset (recorded retrospectively) was used, with one exception. For 6.4% of individuals who had an MS or demyelinating event claim in our administrative data occurring before their MS symptom onset date (based on physician, hospital, or prescription dispensation data), we used the earliest administrative MS or demyelinating event claim to establish the date of MS onset (eTable 1 in Supplement 1).^20^

To compare health care use in a general population, we selected a matched cohort with no evidence of MS (ie, had not visited one of the British Columbia MS clinics, had no diagnostic codes for any demyelinating diseases, and had no prescriptions for an MS DMD). Each person with MS was matched by sex, exact birth year, and postal code at the year of MS onset to account for demographic and regional variability in health care access and use,^21^ with up to 5 individuals randomly selected without replacement from the British Columbia general population.^22^ The matched individuals were then assigned the same MS onset date as their matched person with MS. Persons in the MS and matched cohorts were required to have been a resident of British Columbia for at least 90% of the days in each of the 5 years before the onset date until the person with MS first visited the MS clinic. Residency was based on the total days registered each year in the universal provincial health insurance plan.

### Health Care Use Assessment

**Figure 1.**
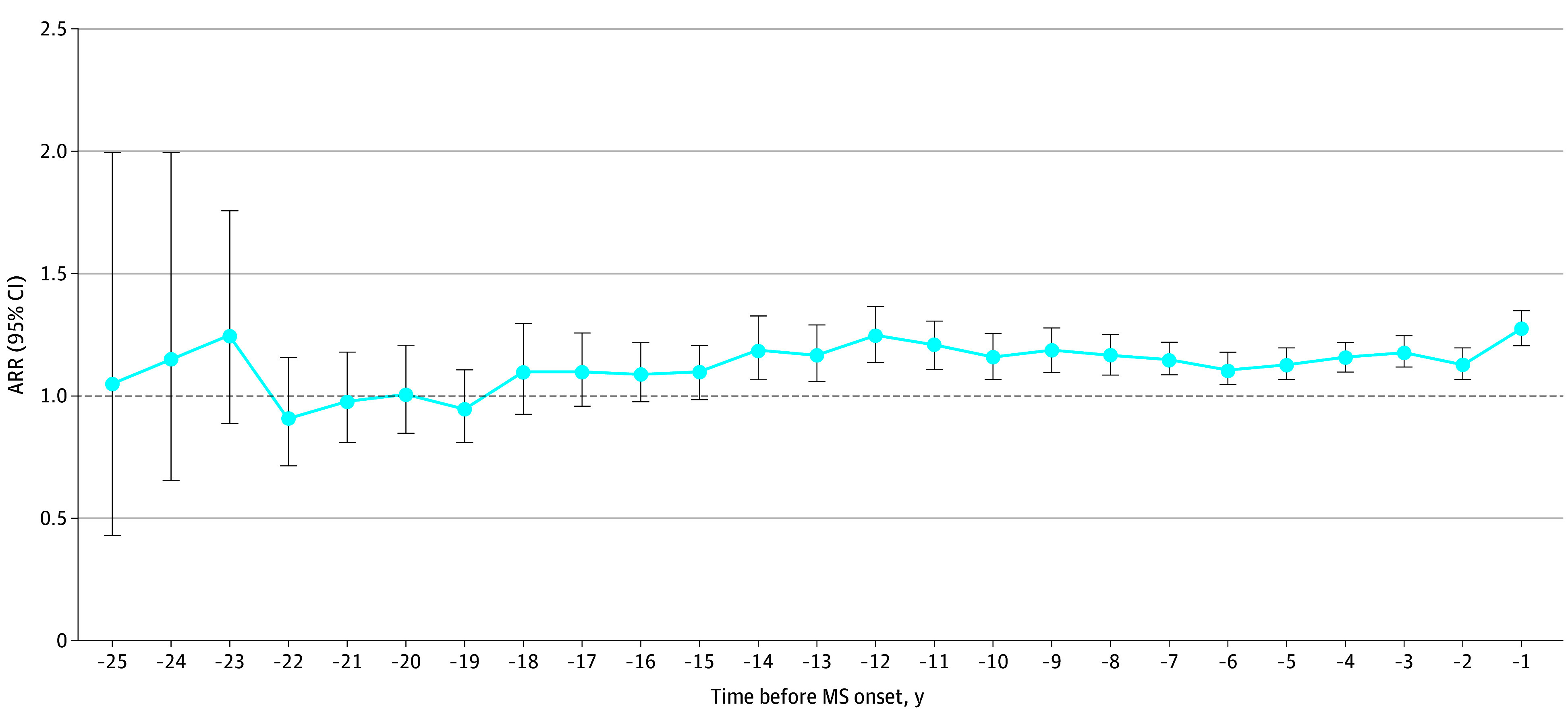
Adjusted Rate Ratios (ARRs) for Physician Visits in the 25 Years Preceding Multiple Sclerosis (MS) Symptom Onset Rate ratios were adjusted by sex, age, socioeconomic status quintile (287 individuals [2.3%] were missing data and were assigned to quintile 3), region of residence (6 persons [0.1%] were missing data and were assigned to urban), and calendar year at MS symptom onset.

First, we compared annual rates of physician visits in the 25 years before MS symptom onset vs the matched general population cohort. Then, guided by these results (Figure 1), we focused on the 15 years before MS symptom onset, in which we observed a consistent divergence in health care use between the cohorts. We examined the difference in annual visit rates by *ICD-9* chapter (grouped according to the World Health Organization’s classification system structure) and by physician specialties (eTables 2 and 3 in Supplement 1) during this period. Finally, to gain deeper insights, we conducted a complementary analysis targeting the specific 3-digit *ICD-9* codes and British Columbia Diagnosis Codes in the 5 years before MS onset. Five years was chosen to capitalize on the consistent (and high number) of persons with MS and matched individuals during this period.

### Statistical Analysis

We used negative binomial regression models to account for overdispersion and generalized estimating equations with an exchangeable working correlation structure to account for within-person correlation.^23^ Models were adjusted for sex and at MS symptom onset, age (continuous), socioeconomic status quintiles, region based on postal codes (urban or rural), and calendar year (continuous). To account for varying follow-up durations, we included person-years as a model offset. An interaction between MS status and the year before the MS onset date was included to trace how health care use changed over time as individuals approached the onset of MS symptoms. Findings are reported as adjusted rate ratios (ARRs) with 95% CIs.

For the crude rate ratios (RRs) of physician visits by *ICD-9* chapters, specialties, and specific 3-digit *ICD-9* codes (and British Columbia Diagnosis Codes), we used an overdispersed Poisson regression model with person-time as the model offset. Findings are reported as RRs with 95% CIs. Results were considered significant if the 95% CI did not include 1. Analyses were conducted using R, version 3.1.0 (R Foundation for Statistical Computing) and SAS, version 4.0.5 (SAS Institute Inc).

## Results

We identified 2038 patients with MS (mean [SD] age at symptom onset, 37.9 [10.9] years, 1508 female [74.0%] and 530 male [26.0%]) and 10 182 matched individuals (Table). Individuals with MS consistently exhibited elevated physician visit ARRs beginning 14 years before MS onset (1.19; 95% CI, 1.07-1.33), peaking at 1.28 (95% CI, 1.21-1.35) in the year immediately preceding MS symptom onset (Figure 1).

**Table.** Cohort Characteristics at the Date of MS Symptom Onset

Characteristic^a^	Individuals, No. (%)		SMD (95% CI)^b^
	MS cohort (n = 2038)	Matched cohort without MS (n = 10 182)	
Sex			
Female	1508 (74.0)	7534 (74.0)	0
Male	530 (26.0)	2648 (26.0)	0
Age, mean (SD), y	37.9 (10.9)	37.9 (10.9)	0
Age group, y			
<30	514 (25.2)	2560 (25.1)	0
30 to <50	1250 (61.3)	6243 (61.3)	0
≥50	274 (13.4)	1379 (13.5)	−0.04
Year of MS symptom onset			
1995-2000	795 (39.0)	3970 (39.0)	0
2001-2010	935 (45.9)	4673 (45.9)	0
2011-2018	308 (15.1)	1539 (15.1)	0
Socioeconomic status quintile^c^^,^^d^			
1 (Lowest)	327 (16.0)	1663 (16.3)	−0.14
2	359 (17.6)	1841 (18.1)	−0.23
3	438 (21.5)	2057 (20.2)	0.60
4	443 (21.7)	2158 (21.2)	0.23
5 (Highest)	429 (21.1)	2218 (21.8)	−0.33
Missing	42 (2.1)	245 (2.4)	−0.12
Region of residence^d^			
Urban	1784 (87.5)	8833 (86.8)	0.81
Rural	254 (12.5)	1343 (13.2)	−0.31
Unknown	0	6 (0.1)	NA

Abbreviations: MS, multiple sclerosis; NA, not applicable; SMD, standardized mean difference.

^a^ For 131 patients (6.4%), the date of an *International Classification of Diseases* code for an earlier demyelinating event was substituted for the neurology-recorded MS symptom onset as the onset date.

^b^ Estimates are shown where relevant.

^c^ Based on each individual’s neighborhood-level income.

^d^ The small number of individuals with missing socioeconomic status were assigned to quintile 3, and those with missing region of residence were assigned to urban.

**Figure 2.**
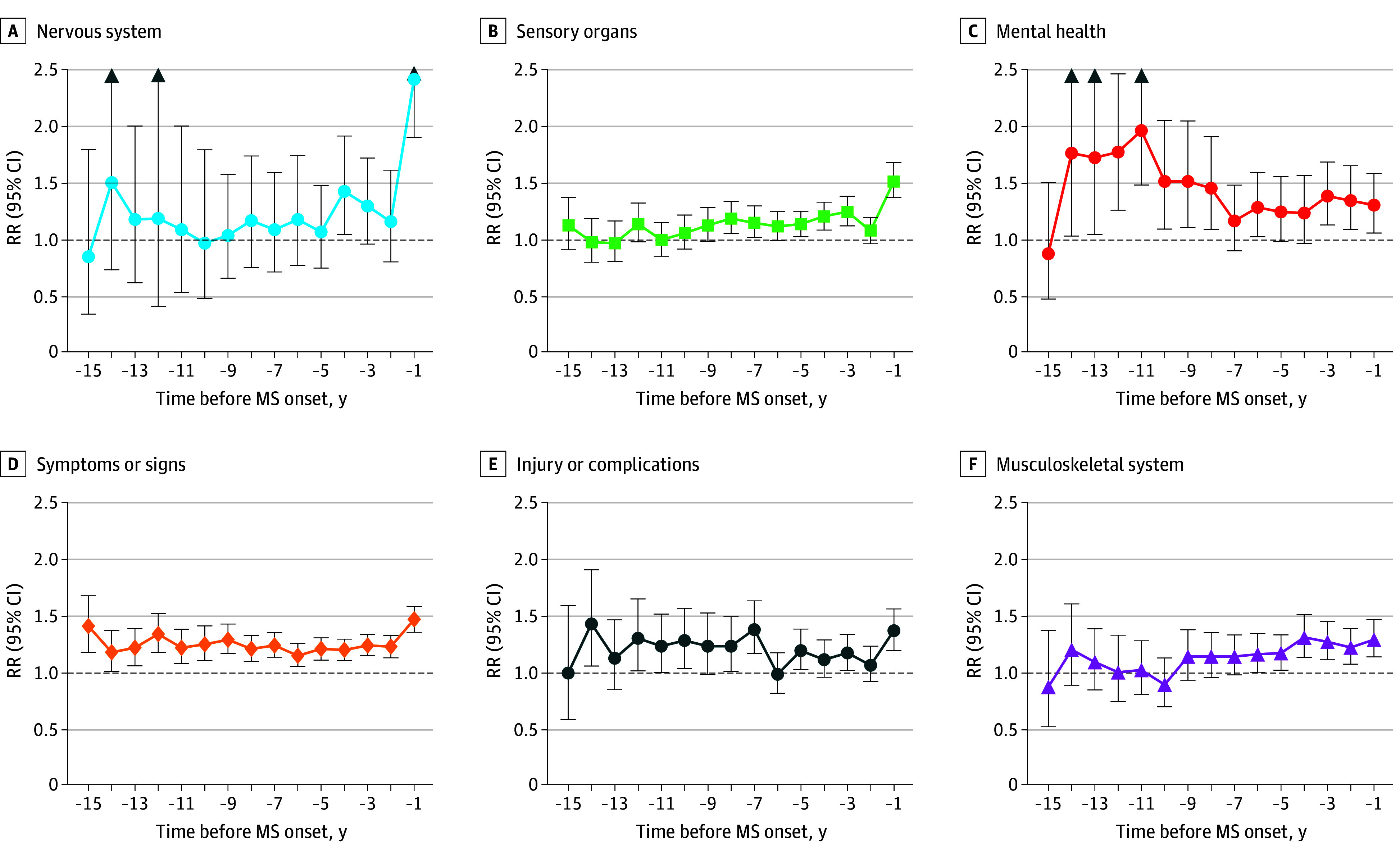
Physician Visits by *International Classification of Diseases, Ninth Revision* (*ICD-9*) Chapter in the 15 Years Preceding Multiple Sclerosis (MS) Symptom Onset Arrows indicate that the upper confidence interval is outside the range of the plot. Additional visits by *ICD-9* chapter are shown in eFigure 2 in Supplement 1. Only *ICD-9* chapters with more than 10 events (MS cohort and the matched cohort without MS combined) across all years in the assessed period were plotted. RR indicates rate ratio.

Findings for the physician visits by *ICD-9* chapter in the 15 years leading up to MS symptom onset vs the matched cohort without MS are shown in Figure 2, eFigure 2 in Supplement 1, and eTable 4 in Supplement 2. Mental health–related physician visit RRs were among those elevated for the longest period before MS onset, being statistically significant at year 14 before onset (1.76; 95% CI, 1.03-2.89) and for nearly all years before onset (excluding years 7, 5, and 4), with the 3 years before MS onset ranging from 1.30 (95% CI, 1.05-1.58) to 1.38 (95% CI, 1.12-1.68). Also elevated for an extended period were visit RRs for ill-defined symptoms and signs, which consistently exceeded 1.15 for 15 years before MS onset, and injury-related visits, which were significantly higher up to 14 years pre-onset, peaking the year before onset (1.47; 95% CI, 1.36-1.58).

Sensory organ–related RRs were elevated from 8 years before onset (1.18; 95% CI, 1.05-1.33), peaking in the year before MS onset (1.51; 95% CI, 1.36-1.68). Musculoskeletal-related visit RRs were consistently elevated from year 5 before onset (1.17; 95% CI, 1.02-1.33), reaching 1.29 (95% CI, 1.14-1.47) in the year before MS onset. Visits for nervous system conditions had significantly elevated RRs at year 4 before onset (1.42; 95% CI, 1.04-1.91) and in the year before MS onset (2.42; 95% CI, 1.90-3.07).

The RRs for the remaining physician visits, such as for blood-related and endocrine conditions, were variable. For example, blood-related visit RRs were significantly elevated at years 10 (3.03; 95% CI, 1.94-4.67) and 9 (1.93; 95% CI, 1.18-3.06) before MS onset, and although they were elevated in some other years before onset, they were not statistically significant. For endocrine visits, the RR was significantly elevated only in year 9 before MS onset (2.20; 95% CI, 1.46-3.25). Pregnancy and childbirth-related visit RRs (female individuals only) also fluctuated in the years leading up to MS onset, with the RRs being less than 1 in the 5 years before MS onset and statistically significant in years 5 (0.71; 95% CI, 0.49-0.99) and 2 (0.68; 95% CI, 0.46-0.97) before onset. While RRs fluctuated before MS onset for the remaining *ICD-9* chapters (visits for circulatory, digestive, genitourinary, infection, respiratory, and skin-related conditions and for neoplasms), no remarkable patterns were observed.

**Figure 3.**
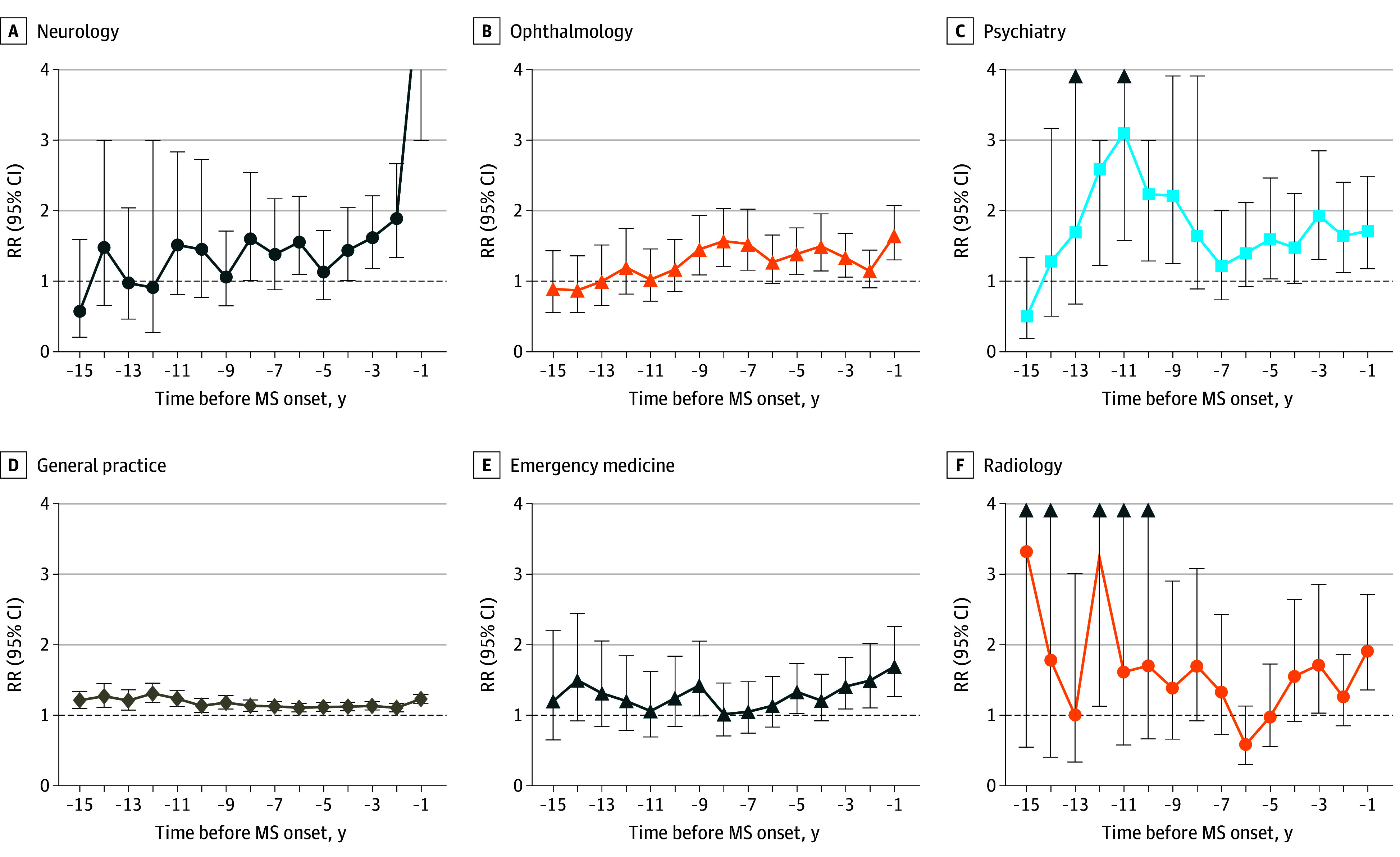
Physician Visits by Specialty in the 15 Years Preceding Multiple Sclerosis (MS) Symptom Onset Arrows indicate that the upper confidence interval is outside the range of the plot. Only specialties with more than 10 events (MS cohort and the matched cohort without MS combined) across all years in the assessed period were plotted (accordingly, neuropsychiatry, cardiology, endocrinology, gastroenterology, and nephrology are not shown). A list of assessed specialties is provided in eTable 3 in Supplement 1. Additional specialties are shown in eFigure 3 in Supplement 1. RR indicates rate ratio.

Physician visit RRs by specialty in the years leading up to MS onset are shown in Figure 3, eFigure 3 in Supplement 1, and eTable 5 in Supplement 2. General practice visit RRs were significantly elevated in each of the 15 years before MS onset, reaching 1.23 (95% CI, 1.17-1.30) in the year before onset. Psychiatry visit RRs showed a significant rise beginning 12 years before symptom onset (2.59; 95% CI, 1.23-5.47). The RRs remained significantly elevated in nearly all subsequent years (except years 8 to 6 and 4 before onset) and ranged from 1.64 (95% CI, 1.12-2.41) to 1.93 (95% CI, 1.31-2.85) in the 3 years before MS onset. Ophthalmology visit RRs were significantly elevated up to 9 years before MS onset (1.45; 95% CI, 1.09-1.94) (except years 6 and 2), peaking at 1.64 (95% CI, 1.30-2.08) in the year before onset. Neurology visit RRs were significantly higher up to 8 years before MS onset (1.61; 95% CI, 1.01-2.55) and were consistently elevated in each of the years before onset (except years 7 and 5), sharply rising to a peak of 5.46 (95% CI, 4.30-6.93) in the year before onset.

Other specialty-related RRs rose closer to MS onset. For example, emergency medicine visit RRs were significantly higher up to 5 years before onset, peaking at 1.69 (95% CI, 1.27-2.26) in the year before MS onset. Radiology visit RRs were significantly elevated in year 3 before onset (1.71; 95% CI, 1.03-2.86), reaching a peak of 1.92 (95% CI, 1.36-2.72) in the year before MS onset. For internal medicine, the RR was significantly elevated only in the year before onset (1.38; 95% CI, 1.08-1.77). Visit RRs for the surgery specialties and orthopedics fluctuated. For example, neurosurgery peaked at 2.65 (95% CI, 1.42-4.96) in the year before MS onset. While visits to other specialties (eg, urology, otolaryngology) exhibited elevated RRs, none differed significantly between cohorts, and no remarkable differences were found for visits to dermatology, obstetrics and gynecology (female individuals only), physical medicine and rehabilitation, or rheumatology. eFigure 4 in Supplement 1 provides an infographic summary of health care use 14 to 15 years before MS symptom onset.

Physician visit RRs by 3-digit *ICD-9 *codes and British Columbia Diagnosis Codes in the 5 years before MS onset are shown in eTable 6 in Supplement 1. By clinical domain, psychiatric-related issues, ie, depressive and neurotic disorders (*ICD-9* codes 311 and 300, respectively), were significantly elevated in the 4 to 5 years before MS onset, with RRs ranging from 1.42 (95% CI, 1.10-1.84) to 2.34 (95% CI, 1.63-3.34). Anxiety and depression (British Columbia Diagnosis Code 50B) also was significant during this time, with RRs in years 4, 3, and 2 before onset ranging from 1.28 (95% CI, 1.01-1.62) to 1.61 (95% CI, 1.23-2.11). Several neurologic conditions were significantly elevated in the year before MS onset, including migraine (*ICD-9* code 346) (RR, 1.85; 95% CI, 1.28-2.67) and other brain or nervous system–related issues (*ICD-9* codes 348 and 349), with RRs ranging from 3.87 (95% CI, 2.55-5.87) to 5.00 (95% CI, 2.25-11.11). Dizziness, vertigo, or insomnia (British Columbia Diagnosis Code 01A) were significantly elevated in the 2 years before onset, with RRs ranging from 1.64 (95% CI, 1.13-2.38) to 1.99 (95% CI, 1.43-2.78). Visual disturbances (*ICD-9* code 368) were significantly elevated in 4 of the 5 years before onset, with the RR peaking at 3.47 (95% CI, 2.67-4.51) the year before MS onset. The RRs for refraction and accommodation (*ICD-9* code 367), strabismus or binocular eye movement (*ICD-9* code 378), disorders of the globe (*ICD-9* code 360), and retinal detachments or defects (*ICD-9* code 361) were elevated in the year before MS onset, ranging from 1.18 (95% CI, 1.02-1.37) to 4.24 (95% CI, 2.26-7.95). Musculoskeletal-related conditions, particularly osteoarthrosis or allied disorders (*ICD-9* code 715) and internal knee derangement (*ICD-9* code 717), were elevated in the year before MS onset, with RRs of 1.95 (95% CI, 1.29-2.94) and 1.81 (95% CI, 1.08-3.05), respectively.

## Discussion

In this matched cohort study, we examined health care use up to 25 years before the clinically determined date of MS symptom onset. In doing so, we observed a sustained rise in all-cause physician visits starting 14 years before MS symptom onset, peaking in the year before onset. We also examined the diagnoses linked to each visit and the physician specialties consulted. We found that elevated rates of mental health–related issues and visits to psychiatrists, as well as ill-defined symptoms and signs and general practice visits, began as early as 15 years before MS symptom onset, preceding the rise in visits related to nervous system issues or consultations with neurologists by approximately 7 to 11 years. Combined, our findings suggest that MS may start earlier than previously thought, with mental health and psychiatric issues among the earliest features of the prodromal period (eFigure 4 in Supplement 1).

By extending the observation window to 25 years before MS onset, our study provides novel insights beyond the typical 5- to 10-year time frame.^6,7,8,11,14,15,24^ By anchoring our analyses to MS symptom onset rather than the first demyelinating event or diagnosis, we ensured a more precise reflection of the early disease course, minimizing biases introduced by diagnostic delays or evolving diagnostic criteria. In addition, the detailed examination of the physician visits offers a more comprehensive understanding of the early stages of MS^25,26,27^ and long-term health care use patterns before MS symptom onset. Biological evidence has suggested that underlying neuroaxonal damage and neurodegeneration may be evident at least 10 years before MS symptom onset, measurable as increases in serum neurofilament light chain (sNfL) levels.^28^ The observed rises in physician visits up to 14 years before MS symptom onset may reflect the lack of specificity of sNfL alone. Recent work has shown that in clinical trial participants with MS, rises in sNfL were preceded by radiologic evidence of MS disease activity, such as gadolinium-enhancing lesions.^29^ Radiologic activity could also occur independent of substantial rises in sNfL.^29^ As peripheral immune activation is thought to occur before central nervous system (CNS) involvement,^30^ it is conceivable that some early symptoms might be due to immune responses outside the CNS and may not be fully captured by sNfL levels at the initial stages of the disease.^13,15^ Visits for mental health conditions and ill-defined symptoms and signs increased 14 to 15 years before MS onset. Previous studies have shown elevations in health care use for depressive, anxiety, and psychiatric conditions, as well as for more nonspecific issues, before MS onset.^11,12,24,31^ However, these studies either focused on a limited time (5 years) before MS onset^12^ or could only access primary care (general practice) visits and the period before the first MS record rather than the clinician-assigned date of MS symptom onset.^11^ By examining a much longer period and before MS symptom onset, we observed a 159% increase in psychiatrist consultations up to 12 years before MS onset and a 76% increase in mental health visits up to 14 years before onset. These early rises may have long-term consequences for patients with MS as psychiatric issues affect disability progression^32,33^ and are associated with increased mortality risk.^34^ The early increase in psychiatric visits may indicate the earliest stages of MS-related immune dysregulation, characterized by elevated proinflammatory cytokines (eg, interleukin 6, tumor necrosis factor α) and blood-brain barrier dysfunction,^35,36^ which may disrupt mood-regulating neurocircuitry.^37^ Similar patterns are observed in other immune-mediated diseases, such as systemic lupus erythematosus and inflammatory arthritis, in which neuropsychiatric symptoms may precede overt clinical manifestations.^38,39^ In neurodegenerative conditions, such as Parkinson disease, depression, and anxiety, the prodromal phase has been linked to early neuroinflammation.^40,41^ These parallels suggest that psychiatric symptoms in MS may serve as early indicators of underlying immune and inflammatory processes.^42,43^

Next to rise were visits for sensory, musculoskeletal, and nervous system conditions 8 to 4 years before MS onset. These issues are commonly observed after MS onset and may, even in the preonset phase, reflect early immunologic or inflammatory changes or emerging CNS demyelination.^7^ The rise in visits for issues such as those related to the sensory system may reflect early visual disturbances, a common symptom of MS, and were reported as higher before an MS or a CNS-related demyelinating diagnosis in other studies.^6,11,13,44^ Musculoskeletal conditions, including osteoarthrosis, joint, and back disorders, are similarly common after MS onset and can be challenging to assess, highlighted by the lack of specific clinical tools in the MS context.^45^ Visits across multiple specialties before MS symptom onset complemented the patterns observed in our *ICD-9* chapter analyses. The diversity of specialties consulted may further illustrate the complexity of early MS presentation. General practice visits were elevated up to 15 years before onset, followed by emergency medicine visits, which were elevated up to 5 years before onset, and radiology visits (3 years before onset). Internal medicine and neurosurgery visits rose only in the year before MS onset. The sequential rises across specialties before MS symptom onset, such as radiology visits, may reflect increased suspicion of underlying neurologic issues requiring additional tests, including imaging analyses.

### Strengths and Limitations

The strength of this study was the use of a large, clinical cohort with linked prospectively accrued health care data. This design minimized recall bias and enabled a comprehensive analysis of physician visits, including by medical specialty and *ICD*-*9*–based diagnoses before MS onset.

The study also had several limitations. The administrative data lacked detailed clinical information and only captured issues that prompted individuals to seek medical care. While health administrative data have been validated for use in patients with MS and other populations,^46^ miscoding or misclassification may occur. However, missingness in our data was low and did not meaningfully differ between cohorts across key variables, such as socioeconomic status and rural or urban residence. Only a small percentage of individuals had an earlier *ICD* code for a demyelinating event, but determining MS symptom onset may be influenced by recall bias and challenges with medical history taking. While elevated neurology and ophthalmology visits may partly reflect a missed opportunity for earlier diagnosis, this seems less likely in our cohort, in which symptom onset was retrospectively determined by neurologists in more than 93% of individuals, supporting the presence of a prolonged prodromal phase.

Efforts to optimize clinical pathways are essential to facilitate earlier recognition of MS while minimizing the risks of overdiagnosis or unnecessary concern for patients and families. Advances in MS diagnosis, including neuroimaging (eg, central vein signs, paramagnetic rim lesions on magnetic resonance imaging)^47,48^ and biochemical biomarkers (eg, cerebrospinal fluid κ-free light chains or sNfL)^49,50^ have shown promise in identifying at-risk individuals.^51^ Our findings may inform future research that integrates clinical, biological, and lifestyle data to enhance strategies for early detection and intervention.^25^ The findings may provide insight into the underlying pathologic processes preceding classical presentation of MS.

## Conclusions

In this matched cohort study of people with and without MS, health care use was higher among those with MS, with a rise in physician visits beginning 14 to 15 years before MS symptom onset, visits for ill-defined symptoms and signs emerging as early as 15 years before onset, and mental health–related visits starting at 14 years before onset (eFigure 4 in Supplement 1). In contrast, nervous system–related visits and neurologist consultations only became significantly elevated 4 to 8 years before MS symptom onset, revealing a distinct temporal pattern in health care use. These results suggest that MS may begin earlier than previously thought, extending the known risk period and emphasizing the importance of investigating earlier time frames for potential risk factors or etiologic causes. They also highlight opportunities to recognize and manage MS earlier in its disease course.

## Notes

Supplement 1. **eTable 1.** Multiple Sclerosis–Specific and Demyelinating Disease–Related *International Classification of Diseases, Ninth Revision* (*ICD-9*) and *International Statistical Classification of Diseases, Tenth Revision* (*ICD-10*) Codes and MS Disease-Modifying Drugs Used to Determine the Earliest Possible Onset of MS **eTable 2.** *ICD-9* Chapters and BC Diagnostic Codes Assessed **eTable 3.** List of Physician Specialties Assessed **eTable 6.** Rate Ratios (95% CIs) for *ICD-9*, 3-Digit Codes and BC-Specific Diagnostic Codes by Year in the 5 Years Preceding MS Onset **eFigure 1.** Flowchart Summarizing the Selection of the Multiple Sclerosis (MS) Cohort **eFigure 2.** Physician Visits by *ICD-9* Chapter in the 15 Years Preceding Multiple Sclerosis (MS) Symptom Onset **eFigure 3.** Physician Visits by Specialty in the 15 Years Preceding Multiple Sclerosis Symptom Onset **eFigure 4.** Higher Health Care Use 14 to 15 Years Before Multiple Sclerosis (MS) Symptom Onset: Matched Clinical Cohort Study

Supplement 2. **eTable 4.** Rate Ratios (95% CIs) for Physician Visits by *ICD-9* Chapters for Each year in the 15 Years Preceding MS Onset **eTable 5.** Rate Ratios (95% CIs) for Specialty Visits for Each Year in the 15 Years Preceding MS Onset

Supplement 3.
Data Sharing Statement
