# Error in Abstract Results

**DOI:** 10.1001/jamanetworkopen.2025.34175

**Published:** 2025-08-26

In the Original Investigation titled “Health Care Use Before Multiple Sclerosis Symptom Onset,”^1^ published August 1, 2025, there was an error in the rate ratio reported for ill-defined symptoms and signs the year before multiple sclerosis onset. The rate ratio was corrected to read 1.47 (95% CI, 1.36-1.58). This article has been corrected.^1^
