# Supplementary material for: Health Care Use Before Multiple Sclerosis Symptom Onset
Source: JAMA Netw Open. 2025 Aug 1;8(8):e2524635. doi: 10.1001/jamanetworkopen.2025.24635 (PMC12317355; doi:10.1001/jamanetworkopen.2025.24635)
Supplement: Supplement 1. — eTable 1. Multiple Sclerosis–Specific and Demyelinating Disease–Related International Classification of Diseases, Ninth Revision (ICD-9) and International Statistical Classification of Diseases, Tenth Revision (ICD-10) Codes and MS Disease-Modifying Drugs Used to Determine the Earliest Possible Onset of MS eTable 2. ICD-9 Chapters and BC Diagnostic Codes Assessed eTable 3. List of Physician Specialties Assessed eTable 6. Rate Ratios (95% CIs) for ICD-9, 3-Digit Codes and BC-Specific Diagnostic Codes by Year in the 5 Years Preceding MS Onset eFigure 1. Flowchart Summarizing the Selection of the Multiple Sclerosis (MS) Cohort eFigure 2. Physician Visits by ICD-9 Chapter in the 15 Years Preceding Multiple Sclerosis (MS) Symptom Onset eFigure 3. Physician Visits by Specialty in the 15 Years Preceding Multiple Sclerosis Symptom Onset eFigure 4. Higher Health Care Use 14 to 15 Years Before Multiple Sclerosis (MS) Symptom Onset: Matched Clinical Cohort Study [file jamanetwopen-e2524635-s001.pdf]

Supplemental Online Content

Ruiz-Algueró M, Zhu F, Chertcoff A, Zhao Y, Marrie RA, Tremlett H. Health care use before multiple sclerosis symptom onset. *JAMA Netw Open*. 2025;8(8):e2524635. doi:10.1001/jamanetworkopen.2025.24635

eTable 1. Multiple Sclerosis–Specific and Demyelinating Disease–Related *International Classification of Diseases, Ninth Revision* (*ICD-9*) and *International Statistical Classification of Diseases, Tenth Revision* (*ICD-10*) Codes and MS MS Disease-Modifying Drugs Used to Determine the Earliest Possible Onset of MS

eTable 2. *ICD-9* Chapters and BC Diagnostic Codes Assessed

eTable 3. List of Physician Specialties Assessed

eTable 6. Rate Ratios (95% CIs) for *ICD-9*, 3-Digit Codes and BC-Specific Diagnostic Codes by Year in the 5 Years Preceding MS Onset

eFigure 1. Flowchart Summarizing the Selection of the Multiple Sclerosis (MS) Cohort

eFigure 2. Physician Visits by ICD-9 Chapter in the 15 Years Preceding Multiple Sclerosis (MS) Symptom Onset

eFigure 3. Physician Visits by Specialty in the 15 Years Preceding Multiple Sclerosis Symptom Onset

eFigure 4. Higher Health Care Use 14 to 15 Years Before Multiple Sclerosis (MS) Symptom Onset: Matched Clinical Cohort Study

This supplemental material has been provided by the authors to give readers additional information about their work.

© 2025 Ruiz-Algueró M et al. *JAMA Network Open*.

eTable 1. Multiple Sclerosis–Specific and Demyelinating Disease–Related *International Classification of Diseases, Ninth Revision* (*ICD-9*) and *International Statistical Classification of Diseases, Tenth Revision* (*ICD-10*) Codes and MS MS Disease-Modifying Drugs Used to Determine the Earliest Possible Onset of MS

This affected a minority (n=131, 6.4%) of PwMS for whom an earlier claim-related date was used to replace the clinically determined MS symptom onset date.

eTable 1.1: ICD codes

	**Physician data**	**Hospital data**	
**MS-specific ICD codes and Demyelinating disease codes**	**ICD-9**	**ICD-9**	**ICD-10**
**MS-specific diagnosis (Multiple sclerosis)**	340	340	G35
**Demyelinating disease codes**			
Optic neuritis	377	377.3	H46
Acute transverse myelitis	323<br>341	323.82<br>341.2	G37.3
Acute disseminated encephalomyelitis	323	323	G36.9
Demyelinating disease of CNS unspecified	341	341.9	G37.8
Other acute disseminated demyelination			G36
Neuromyelitis Optica	341	341.0	G36.0

© 2025 Ruiz-Algueró M et al. *JAMA Network Open*.

eTable 1.2. MS disease-modifying drugs and their respective unique drug identification numbers

**MS disease-modifying drugs (generic/therapeutic classes and brand names)**		**Drug identification numbers (assigned by Health Canada)**
Beta-interferon	Betaseron® (0.3 mg/vial)	02169649
	Extavia® (0.3mg/vial)	02337819
	Peginterferon beta 1a [Plegridy®] (125mcg/0.5ml)<br>(94mcg/0.5ml)<br>(63 mcg/0.5ml)<br>(starter pack; 63 µg/0.5ml & 94 µg/0.5ml)	02444399<br>02444380<br>02444372<br>02444402
	Avonex® (30 µG/kit);<br>(30 µG/0.5 ml)	02237770<br>02269201
	Rebif® (initiation pack)<br>(11 µG);<br>(22 µG);<br>(44 µG)<br>(66µG);<br>(132µG)	02281708<br>02237317<br>02237319<br>02237320<br>02318253<br>02318261
Glatiramer acetate	Copaxone® (20mg/1 vial);<br>(20mg/1 ml)<br>(40mg/1ml)<br>Teva-glatiramer acetate<br>(20mg/1ml)<br>(Glatect® (20mg/1 ml)	 02233014<br>02245619<br>02456915<br>02441446<br>02460661
Natalizumab	Tysabri® (300mg/15ml)	02286386
Fingolimod	Gilenya® (0.5mg)<br>Gilenya® (0.25mg)<br>TEVA-fingolimod (0.5mg)<br>TARO-fingolimod (0.5mg)<br>Pandoz fingolimod (0.5mg)<br>Pms-fingolimod (0.5mg)<br>Mylan-fingolimod (0.5mg)	02365480<br>02482533<br>02469561<br>02469618<br>02482606<br>02469782<br>02469715

© 2025 Ruiz-Algueró M et al. *JAMA Network Open*.

	Mar-fingolimod (0.5mg)<br>Jamp fingolimod (0.5mg)<br>Apo-fingolimod (0.5mg)<br>ACH-fingolimod (0.5mg)	02474743<br>02487772<br>02469936<br>02475669
Dimethyl fumarate	Tecfidera® (120 mg capsule)<br>Tecfidera® (240 mg capsule)	02404508<br>02420201
Teriflunomide	Aubagio® (14 mg, tablet, oral)	02416328
Alemtuzumab	Lemtrada® (12 mg/unit solution, intravenous infusion))	02418320
Daclizumab	Zinbryta® (150mg/ml pre-filled syringe);<br>(150mg/ml pre-filled pen)	02459620*<br>02459639*
Ocrelizumab	Ocrevus® (300mg/ml)	02467224
Cladribine	Mavenclad® 10 mg	02470179

Note:* Daclizumab was withdrawn from the global market in March 2018.

© 2025 Ruiz-Algueró M et al. *JAMA Network Open*.

eTable 2. *ICD-9* Chapters and BC Diagnostic Codes Assessed

Number	ICD Chapters	Diagnostic Codes	BC-specific diagnostic codes*	Abbreviated name
1	Infectious and Parasitic Diseases	ICD-9: 001 – 139		Infections
2	Neoplasms	ICD-9: 140 – 239		Neoplasms
3	Endocrine, Nutritional And Metabolic Diseases, and Immunity Disorders	ICD-9: 240 – 279		Endocrine
4	Diseases of the Blood and Blood-Forming Organs	ICD-9: 280– 289		Blood-Related
5	Mental, Behavioral and Neurodevelopmental Disorders	ICD-9: 290 – 319	50B	Mental Health
6	Diseases of the Nervous System	ICD-9: 320 – 359		Nervous System
7	Diseases of the Sense Organs	ICD-9: 360 – 389		Sensory Organs
8	Diseases of the Circulatory System	ICD-9: 390 – 459		Circulatory System
9	Diseases of the Respiratory System	ICD-9: 460 – 519		Respiratory System
10	Diseases of the Digestive System	ICD-9: 520 – 579		Digestive System
11	Diseases of the Genitourinary System	ICD-9: 580 – 629		Genitourinary System
12	Complications of Pregnancy, Childbirth and the Puerperium ^	ICD-9: 630-679		Pregnancy/Childbirth
13	Diseases of the Skin and Subcutaneous Tissue	ICD-9: 680 – 709	35A	Skin-related
14	Diseases of the Musculoskeletal System and Connective Tissue	ICD-9: 710 – 739		Musculoskeletal System
15	Congenital Anomalies	ICD-9: 740-759		Congenital Anomalies
16	Certain Conditions Originating in the Perinatal Period	ICD-9: 760-779		Perinatal Period
17	Symptoms, Signs, And Ill-Defined Conditions	ICD-9: 780 – 799	01A, 02A	Symptoms/Signs
18	Injury and poisoning /Complications of surgical and medical care	ICD-9: 800-999/ ICD-9: E800 – E999		Injury/Complications

Note: ICD: International Classification of Diseases, 9th Revision (ICD-9), *BC specific codes that matched to ICD-9 codes and therefore ICD-chapters. The caret symbol (^) indicates assessed in females only.

© 2025 Ruiz-Algueró M et al. *JAMA Network Open*.

eTable 3. List of Physician Specialties Assessed

Specialty	
1	General Practice
2	Dermatology
3	Neurology
4	Psychiatry
5	Neuropsychiatry
6	Obstetrics and Gynaecology^
7	Ophthalmology
8	Otolaryngology
9	General Surgery
10	Neurosurgery
11	Orthopaedic Surgery
12	Urology
13	Internal Medicine
14	Radiology
15	Physical Medicine & Rehab
16	Cardiology
17	Emergency Medicine
18	Rheumatology
19	Endocrinology
20	Gastroenterology
21	Nephrology

Note: The caret (^) indicates assessed in females only.

© 2025 Ruiz-Algueró M et al. *JAMA Network Open*.

eTable 6. Rate Ratios (95% CIs) for *ICD-9*, 3-Digit Codes and BC-Specific Diagnostic Codes by Year in the 5 Years Preceding MS Onset

		Year before MS onset									
		Year -5		Year -4		Year -3		Year -2		Year -1	
ICD-BC Code	Description	RR	95% CI	RR	95% CI	RR	95% CI	RR	95% CI	RR	95% CI
007	Other protozoal intestinal diseases	2.55	0.65, 10.05	0.25*	0.07, 0.95	0.21*	0.06, 0.67	0.06*	0.01, 0.33	0.20*	0.05, 0.88
009	Ill-defined intestinal infections	0.45*	0.27, 0.73	1.13	0.74, 1.71	1.18	0.76, 1.84	0.92	0.60, 1.42	1.18	0.82, 1.69
041	Bacterial infection in conditions classified elsewhere	0.71	0.14, 3.53	5.45*	1.59, 18.71	4.09*	1.05, 15.93	2.17	0.59, 8.04	3.18*	1.12, 9.02
054	Herpes simplex	0.82	0.44, 1.53	0.86	0.49, 1.54	1.78*	1.11, 2.88	0.86	0.51, 1.44	0.83	0.46, 1.50
070	Viral hepatitis	0.19*	0.04, 0.83	0.50	0.10, 2.53	0.77	0.23, 2.59	0.19*	0.04, 0.84	0.08*	0.01, 0.57
110	Dermatophytosis	0.85	0.44, 1.66	1.07	0.60, 1.89	1.10	0.63, 1.92	0.76	0.40, 1.47	1.68*	1.03, 2.75
112	Candidiasis	0.53*	0.29, 0.97	1.14	0.62, 2.11	0.88	0.46, 1.67	0.61	0.35, 1.08	1.38	0.80, 2.38
211	Benign neoplasm of other parts of digestive system	7.00*	1.48, 33.08	5.00*	1.35, 18.44	0.26	0.03, 2.13	0.26	0.03, 2.05	2.08	0.61, 7.09
216	Benign neoplasm of skin	0.94	0.67, 1.30	1.14	0.83, 1.55	1.44*	1.09, 1.91	1.26	0.88, 1.81	1.96*	1.43, 2.67
221	Benign neoplasm of other female genital organs	0.54	0.14, 2.08	4.44	0.93, 21.18	1.67	0.32, 8.56	3.12*	1.01, 9.61	1.25	0.24, 6.50
238	Neoplasm of uncertain behaviour of other and unspecified sites	1.67	0.28, 10.03	2.73	0.86, 8.63	3.75*	1.11, 12.62	0.59	0.08, 4.59	0.65	0.14, 3.11
245	Thyroiditis	1.67	0.47, 5.91	3.57	0.76, 16.77	6.66*	1.75, 25.34	0.71	0.19, 2.62	2.62	0.92, 7.46
259	Other endocrine disorders	0.89	0.28, 2.81	0.57	0.21, 1.57	1.62	0.77, 3.44	1.12	0.40, 3.18	3.37*	1.27, 8.94
272	Disorders of lipoid metabolism	0.89	0.57, 1.39	0.68	0.37, 1.26	1.13	0.75, 1.71	0.76	0.46, 1.25	0.69*	0.48, 0.99
289	Other diseases of blood and blood-forming organs	0.26*	0.07, 0.96	1.06	0.39, 2.91	0.94	0.37, 2.41	0.80	0.25, 2.53	0.96	0.22, 4.09
298	Other nonorganic psychoses	0.28	0.05, 1.61	0.11*	0.01, 0.89	2.00	0.35, 11.34	0.31	0.03, 2.86	0.42	0.07, 2.53
300	Neurotic disorders	1.54	0.94, 2.55	1.54*	1.01, 2.35	2.34*	1.63, 3.34	1.84*	1.17, 2.89	1.72*	1.08, 2.73

© 2025 Ruiz-Algueró M et al. *JAMA Network Open*.

		Year before MS onset									
		Year -5		Year -4		Year -3		Year -2		Year -1	
ICD-BC Code	Description	RR	95% CI	RR	95% CI	RR	95% CI	RR	95% CI	RR	95% CI
301	Personality disorders	0.40	0.13, 1.26	1.08	0.39, 3.02	0.81	0.25, 2.57	0.27*	0.09, 0.76	0.50	0.19, 1.26
304	Drug dependence	0.27*	0.10, 0.74	0.10*	0.04, 0.23	0.14*	0.04, 0.51	0.44	0.09, 2.05	0.54	0.13, 2.36
305	Nondependent abuse of drugs	2.95*	1.12, 7.80	2.32	0.91, 5.92	1.15	0.47, 2.85	0.37	0.11, 1.26	0.68	0.20, 2.23
310	Specific nonpsychotic mental disorders following organic brain damage	0.05*	0.01, 0.50	0.11*	0.01, 0.99	0.70	0.10, 4.81	0.22	0.04, 1.27	0.53	0.12, 2.25
311	Depressive disorder, not elsewhere classified	1.60*	1.21, 2.12	1.65*	1.24, 2.19	1.63*	1.27, 2.09	1.62*	1.28, 2.04	1.42*	1.10, 1.84
314	Hyperkinetic syndrome of childhood	7.18*	2.05, 25.15	1.08	0.18, 6.48	2.80	0.56, 13.96	3.75*	1.23, 11.46	1.45	0.43, 4.87
346	Migraine	1.24	0.82, 1.88	1.64*	1.09, 2.47	1.09	0.72, 1.65	1.26	0.81, 1.94	1.85*	1.28, 2.67
348	Other conditions of brain	1.09	0.35, 3.38	1.18	0.46, 2.99	2.22	0.88, 5.61	0.91	0.25, 3.34	5.00*	2.25, 11.11
349	Other and unspecified disorders of the nervous system	1.29	0.75, 2.22	0.70	0.38, 1.30	1.37	0.80, 2.35	1.30	0.72, 2.34	3.87*	2.55, 5.87
353	Nerve root and plexus disorders	0.36	0.08, 1.65	1.18	0.48, 2.86	1.15	0.26, 5.18	3.09*	1.07, 8.92	2.88*	1.24, 6.69
354	Mononeuritis of upper limb and mononeuritis multiplex	0.77	0.37, 1.60	1.92	0.80, 4.61	1.36	0.74, 2.48	1.36	0.78, 2.37	2.80*	1.74, 4.51
355	Mononeuritis of lower limb	0.38	0.08, 1.82	1.00	0.29, 3.50	0.53	0.12, 2.36	0.67	0.13, 3.43	3.21*	1.34, 7.70
356	Hereditary and idiopathic peripheral neuropathy	1.67	0.32, 8.80	1.18	0.34, 4.01	3.33*	1.30, 8.54	2.50	0.85, 7.36	5.71*	2.67, 12.21
360	Disorders of the globe	0.62	0.14, 2.80	2.31	0.77, 6.87	0.28	0.04, 2.08	0.83	0.18, 3.78	3.89*	1.28, 11.76
361	Retinal detachments and defects	0.42	0.10, 1.82	1.76	0.67, 4.66	1.15	0.40, 3.35	0.61	0.21, 1.78	3.07*	1.27, 7.44

© 2025 Ruiz-Algueró M et al. *JAMA Network Open*.

		Year before MS onset									
		Year -5		Year -4		Year -3		Year -2		Year -1	
ICD-BC Code	Description	RR	95% CI	RR	95% CI	RR	95% CI	RR	95% CI	RR	95% CI
362	Other retinal disorders	0.77	0.39, 1.54	0.62	0.34, 1.12	0.94	0.56, 1.59	1.08	0.57, 2.05	2.97*	1.92, 4.60
364	Disorders of iris and ciliary body	4.40*	1.74, 11.13	2.50	0.77, 8.06	4.58*	1.77, 11.85	1.10	0.35, 3.49	1.95	0.41, 9.39
366	Cataract	3.00*	1.42, 6.32	1.31	0.64, 2.67	1.35	0.67, 2.72	1.23	0.66, 2.31	1.18	0.6, 2.34
367	Disorders of refraction and accommodation	1.01	0.89, 1.15	1.01	0.88, 1.16	1.11	0.97, 1.27	1.16*	1.01, 1.33	1.18*	1.02, 1.37
368	Visual disturbances	1.50*	1.08, 2.08	2.03*	1.45, 2.84	1.43*	1.06, 1.94	1.31	0.93, 1.86	3.47*	2.67, 4.51
371	Corneal opacity and other disorders of cornea	0.71	0.36, 1.39	0.98	0.44, 2.18	1.18	0.58, 2.42	0.40*	0.19, 0.82	0.78	0.43, 1.43
374	Other disorders of eyelids	1.52	0.71, 3.24	2.65*	1.08, 6.53	0.49	0.12, 2.05	1.39	0.63, 3.05	1.19	0.58, 2.44
378	Strabismus and other disorders of binocular eye movements	2.40*	1.26, 4.58	1.38	0.66, 2.91	2.45*	1.30, 4.62	2.28*	1.03, 5.03	4.24*	2.26, 7.95
379	Other disorders of eye	1.03	0.70, 1.50	0.95	0.66, 1.35	1.12	0.79, 1.58	1.51*	1.09, 2.09	2.63*	2.02, 3.43
381	Nonsuppurative otitis media and eustachian tube disorders	0.77	0.46, 1.27	1.16	0.66, 2.01	1.64	0.97, 2.78	1.09	0.68, 1.76	1.69*	1.04, 2.75
382	Suppurative and unspecified otitis media	1.20	0.88, 1.63	1.52*	1.12, 2.07	1.54*	1.11, 2.13	1.17	0.86, 1.59	0.91	0.63, 1.30
386	Vertiginous syndromes and other disorders of vestibular system	1.37	0.66, 2.84	1.20	0.68, 2.10	1.17	0.65, 2.12	1.21	0.69, 2.10	2.44*	1.51, 3.94
401	Essential hypertension	0.82	0.56, 1.19	0.87	0.62, 1.24	0.70*	0.51, 0.97	0.90	0.64, 1.27	1.02	0.77, 1.37
459	Other disorders of circulatory system	0.83	0.18, 3.79	0.23	0.02, 2.25	2.91	0.58, 14.59	3.75*	1.23, 11.40	2.22	0.51, 9.62
461	Acute sinusitis	1.00	0.78, 1.28	1.22	0.96, 1.56	0.93	0.73, 1.18	1.38*	1.08, 1.75	1.27*	1.01, 1.61
463	Acute tonsillitis	0.88	0.63, 1.24	0.69*	0.49, 0.98	0.98	0.69, 1.39	0.73	0.47, 1.14	0.57*	0.36, 0.91
466	Acute bronchitis and bronchiolitis	1.07	0.84, 1.36	1.12	0.88, 1.41	1.31*	1.05, 1.62	0.97	0.76, 1.23	1.01	0.78, 1.30

© 2025 Ruiz-Algueró M et al. *JAMA Network Open*.

		Year before MS onset									
		Year -5		Year -4		Year -3		Year -2		Year -1	
ICD-BC Code	Description	RR	95% CI	RR	95% CI	RR	95% CI	RR	95% CI	RR	95% CI
470	Deflected nasal septum	2.08	0.81, 5.37	1.12	0.38, 3.28	1.22	0.44, 3.38	3.33*	1.24, 8.93	0.81	0.34, 1.92
472	Chronic pharyngitis and nasopharyngitis	0.73	0.44, 1.22	1.75*	1.04, 2.93	0.78	0.48, 1.25	0.65	0.35, 1.21	1.03	0.64, 1.68
473	Chronic sinusitis	0.74	0.33, 1.68	2.04*	1.20, 3.48	1.51	0.84, 2.72	1.62	0.89, 2.97	1.09	0.51, 2.35
480	Viral pneumonia	0.96	0.23, 4.09	1.11	0.22, 5.62	6.00*	1.23, 29.17	1.67	0.20, 13.60	1.31	0.24, 7.26
485	Bronchopneumonia, organism unspecified	0.60	0.15, 2.40	0.62	0.13, 2.91	0.83	0.22, 3.16	6.99*	1.74, 28.12	0.38	0.05, 3.17
493	Asthma	1.20	0.86, 1.67	1.72*	1.19, 2.48	1.07	0.78, 1.48	1.05	0.72, 1.52	1.10	0.80, 1.53
496	Chronic airways obstruction, not elsewhere classified	0.71	0.20, 2.60	0.11*	0.01, 0.87	0.13	0.02, 1.00	0.64	0.17, 2.45	0.41	0.11, 1.47
519	Other diseases of respiratory system	1.40	0.63, 3.10	3.23*	1.49, 7.00	2.29*	1.14, 4.61	1.51	0.66, 3.43	1.16	0.50, 2.69
524	Dentofacial anomalies, including malocclusion	1.13	0.44, 2.93	0.94	0.27, 3.29	0.45	0.10, 2.14	3.33*	1.36, 8.17	2.80*	1.04, 7.55
530	Diseases of oesophagus	2.12*	1.13, 3.98	0.82	0.40, 1.68	1.42	0.71, 2.82	1.53	0.90, 2.58	1.53	0.80, 2.93
540	Acute appendicitis	5.62*	1.54, 20.47	1.93	0.29, 12.95	6.87*	1.87, 25.26	0.93	0.28, 3.03	4.23*	1.25, 14.26
556	Idiopathic proctocolitis	0.18	0.02, 1.41	2.22	0.77, 6.38	1.12	0.33, 3.83	0.19*	0.05, 0.63	0.25*	0.07, 0.84
564	Functional digestive disorders, not elsewhere classified	1.08	0.57, 2.06	0.94	0.51, 1.70	2.15*	1.20, 3.75	1.23	0.76, 1.97	1.31	0.78, 2.21
565	Anal fissure and fistula	1.51	0.67, 3.44	1.34	0.59, 3.03	1.85	0.69, 4.94	1.76	0.63, 4.88	2.90*	1.28, 6.60
569	Other disorders of intestine	1.77*	1.09, 2.90	1.20	0.67, 2.14	1.44	0.93, 2.23	1.71*	1.11, 2.65	1.19	0.71, 1.98
575	Other disorders of gallbladder	0.27*	0.08, 0.95	0.45	0.14, 1.51	0.68	0.27, 1.73	3.00*	1.18, 7.61	1.32	0.52, 3.34
578	Gastrointestinal haemorrhage	2.34	0.93, 5.91	3.61*	1.24, 10.54	0.44	0.13, 1.51	0.43	0.10, 1.98	1.76	0.46, 6.75
596	Other disorders of bladder	1.27	0.60, 2.71	0.49	0.12, 1.95	2.55*	1.15, 5.65	1.87	0.91, 3.86	2.62*	1.33, 5.13

© 2025 Ruiz-Algueró M et al. *JAMA Network Open*.

		Year before MS onset									
		Year -5		Year -4		Year -3		Year -2		Year -1	
ICD-BC Code	Description	RR	95% CI	RR	95% CI	RR	95% CI	RR	95% CI	RR	95% CI
599	Other disorders of urethra and urinary tract	1.46	0.99, 2.15	1.21	0.88, 1.68	1.62*	1.19, 2.20	1.22	0.84, 1.76	1.23	0.88, 1.72
600	Hyperplasia of prostrate	2.78	0.68, 11.39	2.27	0.5, 10.3	4.12*	1.45, 11.71	1.07	0.37, 3.09	0.58	0.12, 2.67
606	Infertility, male	0.22*	0.05, 0.94	0.74	0.2, 2.73	1.11	0.3, 4.18	0.67	0.13, 3.38	1.25	0.44, 3.56
608	Other disorders of male genital organs#	1.16	0.51, 2.67	0.68	0.22, 2.12	0.74	0.30, 1.86	2.56*	1.34, 4.89	1.48	0.69, 3.18
610	Benign mammary dysplasias^	1.77*	1.2, 2.61	1.06	0.62, 1.80	1.06	0.67, 1.67	1.35	0.86, 2.11	1.15	0.65, 2.04
620	Noninflammatory disorders of ovary, fallopian tube and broad ligament^	0.82	0.38, 1.79	0.80	0.29, 2.22	0.85	0.39, 1.83	2.19*	1.12, 4.26	1.35	0.61, 2.95
625	Pain and other symptoms associated with female genital organs^	1.59*	1.17, 2.14	1.08	0.80, 1.47	1.17	0.87, 1.58	1.21	0.83, 1.76	1.17	0.81, 1.68
626	Disorders of menstruation and other abnormal bleeding from female^	0.84	0.71, 1.00	1.04	0.89, 1.22	1.19*	1.01, 1.41	1.07	0.90, 1.26	0.94	0.78, 1.13
632	Missed abortion	1.07	0.38, 2.98	1.38	0.52, 3.67	1.04	0.29, 3.8	0.24	0.05, 1.09	0.29*	0.09, 0.99
633	Ectopic pregnancy	0.58	0.11, 2.91	2.31	0.54, 9.84	0.00*	0.00, 0.00	0.62	0.16, 2.4	0.31	0.04, 2.32
646	Other complications of pregnancy, not elsewhere classified^	0.80	0.43, 1.49	0.9	0.54, 1.5	0.52*	0.31, 0.88	0.61	0.31, 1.21	0.91	0.46, 1.82
650	Delivery in a completely normal case^	0.52*	0.33, 0.81	0.75	0.50, 1.14	0.94	0.57, 1.55	0.79	0.45, 1.41	1.11	0.74, 1.68
676	Other disorders of the breast associated with childbirth, and disorders	0.13*	0.02, 0.97	0.17	0.02, 1.3	0.37	0.09, 1.59	0.56	0.15, 2.01	0.76	0.22, 2.59
681	Cellulitis and abscess of finger and toe	0.80	0.31, 2.05	1.18	0.53, 2.62	0.53	0.18, 1.55	1.21	0.59, 2.47	2.22*	1.12, 4.41

© 2025 Ruiz-Algueró M et al. *JAMA Network Open*.

		Year before MS onset									
		Year -5		Year -4		Year -3		Year -2		Year -1	
ICD-BC Code	Description	RR	95% CI	RR	95% CI	RR	95% CI	RR	95% CI	RR	95% CI
682	Other cellulitis and abscess	1.07	0.73, 1.56	0.99	0.70, 1.41	1.48*	1.03, 2.11	0.96	0.68, 1.35	0.89	0.62, 1.28
692	Contact dermatitis and other eczema	0.62*	0.45, 0.87	1.12	0.81, 1.55	0.76	0.53, 1.10	1.05	0.79, 1.39	1.08	0.81, 1.45
698	Pruritis and related conditions	0.68	0.15, 3.18	3.87*	1.29, 11.61	0.91	0.27, 3.00	0.66	0.21, 2.06	0.41	0.12, 1.41
700	Corns and callosities	0.65	0.22, 1.91	1.65	0.58, 4.65	2.80*	1.01, 7.75	2.16	0.77, 6.18	2.59	0.92, 7.28
701	Other hypertrophic and atrophic conditions of skin	1.37	0.57, 3.31	0.50	0.19, 1.31	0.86	0.38, 1.92	1.14	0.47, 2.73	0.40*	0.17, 0.97
706	Diseases of sebaceous glands	0.63*	0.42, 0.95	1.00	0.69, 1.45	0.94	0.64, 1.38	0.72	0.50, 1.04	1.02	0.70, 1.48
714	Rheumatoid arthritis and other inflammatory polyarthropathies	0.48	0.20, 1.16	0.34*	0.13, 0.86	0.67	0.25, 1.81	0.38*	0.18, 0.78	1.01	0.44, 2.33
715	Osteoarthrosis and allied disorders	1.11	0.62, 2.00	0.76	0.43, 1.33	1.06	0.69, 1.62	1.21	0.81, 1.83	1.95*	1.29, 2.94
717	Internal derangement of knee	1.10	0.64, 1.88	0.87	0.51, 1.50	0.96	0.56, 1.64	1.25	0.76, 2.05	1.81*	1.08, 3.05
719	Other and unspecified disorder of joint	1.05	0.75, 1.47	0.85	0.57, 1.27	1.15	0.81, 1.63	0.83	0.55, 1.25	1.66*	1.09, 2.52
720	Ankylosing spondylitis and other inflammatory spondylopathies	1.19	0.33, 4.31	4.00*	1.01, 15.75	0.50	0.06, 4.12	1.15	0.23, 5.72	1.39	0.32, 6.04
724	Other and unspecified disorders of back	1.26*	1.04, 1.52	1.57*	1.27, 1.93	1.32*	1.09, 1.59	1.23*	1.02, 1.48	1.37*	1.12, 1.68
727	Other disorders of synovium, tendon and bursa	0.95	0.72, 1.25	0.90	0.67, 1.21	1.05	0.78, 1.40	1.30	0.97, 1.73	1.27*	1.01, 1.61
728	Disorders of muscle, ligament and fascia	1.48*	1.01, 2.18	1.33	0.87, 2.01	1.91*	1.25, 2.92	1.56*	1.04, 2.34	1.78*	1.22, 2.61
729	Other disorders of soft tissues	1.46	0.99, 2.15	1.63*	1.21, 2.22	1.42	0.98, 2.05	1.21	0.83, 1.76	1.55*	1.11, 2.17

© 2025 Ruiz-Algueró M et al. *JAMA Network Open*.

		Year before MS onset									
		Year -5		Year -4		Year -3		Year -2		Year -1	
ICD-BC Code	Description	RR	95% CI	RR	95% CI	RR	95% CI	RR	95% CI	RR	95% CI
735	Acquired deformities of toe	2.50	0.79, 7.95	3.63*	1.41, 9.35	1.84	0.78, 4.35	1.30	0.54, 3.17	1.62	0.49, 5.31
739	Nonallopathic lesions, not elsewhere classified	1.10	0.88, 1.39	1.32*	1.04, 1.68	1.27*	1.01, 1.59	1.31*	1.04, 1.64	1.19	0.96, 1.48
742	Other congenital anomalies of nervous system	1.04	0.19, 5.79	0.10*	0.01, 0.77	0.53	0.06, 4.28	0.22	0.04, 1.09	0.19	0.02, 1.78
762	Fetus or newborn affected by complications of placenta, cord	1.11	0.24, 5.14	3.33*	1.03, 10.75	1.82	0.48, 6.92	0.50	0.06, 3.90	1.15	0.32, 4.19
780	General symptoms	1.19*	1.07, 1.33	1.13*	1.00, 1.28	1.15*	1.02, 1.29	1.23*	1.10, 1.39	1.43*	1.29, 1.60
781	Symptoms involving nervous and musculoskeletal systems	1.30*	1.08, 1.57	1.25*	1.06, 1.48	1.53*	1.31, 1.80	1.36*	1.14, 1.63	2.20*	1.87, 2.59
782	Symptoms involving skin and other integumentary tissue	1.04	0.84, 1.27	1.19*	1.00, 1.41	1.19*	1.02, 1.40	1.16	0.99, 1.37	1.29*	1.11, 1.49
784	Symptoms involving head and neck	1.27*	1.04, 1.55	1.21*	1.00, 1.47	1.28*	1.05, 1.56	1.33*	1.07, 1.65	1.72*	1.43, 2.06
786	Symptoms involving respiratory system and other chest symptoms	1.38*	1.14, 1.67	1.28*	1.04, 1.56	1.40*	1.14, 1.70	1.13	0.94, 1.35	1.13	0.93, 1.39
788	Symptoms involving urinary system	1.34	0.99, 1.80	1.50*	1.14, 1.98	1.30	0.97, 1.72	1.46*	1.10, 1.94	1.13	0.84, 1.52
789	Other symptoms involving abdomen and pelvis	1.39*	1.12, 1.73	1.21	0.96, 1.52	1.12	0.92, 1.36	1.21	0.96, 1.52	1.21	0.95, 1.54
790	Nonspecific findings on examination of blood	1.00	0.43, 2.32	1.84*	1.01, 3.32	0.89	0.32, 2.47	0.82	0.40, 1.66	1.15	0.59, 2.23
795	Nonspecific abnormal histological and	0.85	0.37, 1.93	0.97	0.38, 2.48	0.29*	0.08, 0.99	0.68	0.28, 1.68	0.58	0.18, 1.84

© 2025 Ruiz-Algueró M et al. *JAMA Network Open*.

		Year before MS onset									
		Year -5		Year -4		Year -3		Year -2		Year -1	
ICD-BC Code	Description	RR	95% CI	RR	95% CI	RR	95% CI	RR	95% CI	RR	95% CI
	immunological findings										
813	Fracture of radius and ulna	0.75	0.12, 4.63	1.13	0.4, 3.16	2.98*	1.21, 7.32	0.99	0.44, 2.20	0.53	0.22, 1.31
823	Fracture of tibia and fibula	5.00*	1.68, 14.89	2.6	0.9, 7.51	3.54*	1.15, 10.92	2.66	0.89, 7.94	1.31	0.27, 6.40
824	Fracture of ankle	4.66*	1.88, 11.58	2.02	0.82, 4.98	1.30	0.51, 3.32	0.45	0.08, 2.54	0.95	0.33, 2.73
827	Other, multiple and ill-defined fractures of lower limb	0.79	0.14, 4.32	1.07	0.28, 4.14	7.99*	2.05, 31.11	0.29	0.04, 2.31	0.91	0.11, 7.37
840	Sprains and strains of shoulder and upper arm	1.12	0.78, 1.60	1.31	0.93, 1.84	1.16	0.84, 1.60	1.31	0.90, 1.91	1.40*	1.01, 1.95
845	Sprains and strains of ankle and foot	1.58*	1.07, 2.33	1.09	0.75, 1.60	1.01	0.68, 1.49	0.68*	0.48, 0.96	1.27	0.80, 2.01
846	Sprains and strains of sacroiliac region	1.39	0.81, 2.37	0.73	0.41, 1.30	0.96	0.52, 1.78	0.50*	0.26, 0.97	1.30	0.74, 2.28
847	Sprains and strains of other and unspecified parts of back	1.03	0.72, 1.48	0.87	0.61, 1.24	1.11	0.82, 1.49	0.99	0.73, 1.34	1.55*	1.08, 2.21
879	Open wound of other and unspecified sites, except limbs	1.37	0.85, 2.22	0.91	0.56, 1.50	0.69	0.39, 1.23	1.95*	1.23, 3.07	1.26	0.75, 2.12
916	Superficial injury of hip, thigh, leg and ankle	0.91	0.31, 2.65	1.43	0.54, 3.77	5.58*	1.38, 22.63	2.40	0.78, 7.41	1.87	0.61, 5.76
917	Superficial injury of foot and toe(s)	0.64	0.22, 1.87	1.46	0.52, 4.08	2.64*	1.06, 6.62	5.45*	2.01, 14.80	2.50	0.85, 7.37
918	Superficial injury of eye and adnexa	0.50	0.15, 1.67	1.46	0.53, 4.04	3.07*	1.17, 8.10	0.83	0.28, 2.46	3.00	0.68, 13.24
920	Contusion of face, scalp, and neck except eye(s)	0.75	0.22, 2.60	1.00	0.28, 3.54	1.11	0.37, 3.31	2.64*	1.04, 6.71	1.11	0.37, 3.31
923	Contusion of upper limb	1.18	0.56, 2.49	1.03	0.43, 2.51	1.48	0.67, 3.28	1.02	0.44, 2.38	3.66*	1.34, 10.05

© 2025 Ruiz-Algueró M et al. *JAMA Network Open*.

		Year before MS onset									
		Year -5		Year -4		Year -3		Year -2		Year -1	
ICD-BC Code	Description	RR	95% CI	RR	95% CI	RR	95% CI	RR	95% CI	RR	95% CI
924	Contusion of lower limb and other unspecified sites	1.33	0.81, 2.18	0.95	0.48, 1.90	1.09	0.59, 2.03	1.75	0.77, 3.96	1.82*	1.06, 3.15
995	Certain adverse effects not elsewhere classified	2.70*	1.50, 4.87	2.61*	1.23, 5.52	1.48	0.81, 2.71	0.98	0.61, 1.57	1.73*	1.04, 2.85
995	Certain adverse effects not elsewhere classified	2.7*	1.5, 4.87	2.61*	1.23, 5.52	1.48	0.81, 2.71	0.98	0.61, 1.57	1.73*	1.04, 2.85
999	Complications of medical care, not elsewhere classified	0.96	0.69, 1.35	1.02	0.74, 1.39	1.13	0.83, 1.52	1.27	0.96, 1.68	1.65*	1.27, 2.13
01A	Dizziness, Vertigo, Insomnia	0.72	0.46, 1.14	1.16	0.79, 1.71	1.23	0.84, 1.79	1.64*	1.13, 2.38	1.99*	1.43, 2.78
02A	Abdominal Swelling Not Otherwise Specified or Abdominal Pain	1.14	0.86, 1.52	1.22	0.93, 1.61	1.12	0.80, 1.56	1.46*	1.09, 1.96	1.23	0.85, 1.76
50B	Anxiety/Depression	1.26	1, 1.59	1.48*	1.18, 1.86	1.28*	1.01, 1.62	1.61*	1.23, 2.11	1.27	0.97, 1.66

Note: RR: Rate Ratios; CI: Confidence Interval. The years -1, -2, -3, -4, and -5 correspond to the years preceding the onset of MS symptoms. An asterisk (*) indicates statistically significant results, where the 95% confidence interval does not include 1. The hash symbol (#) denotes findings specific to males only, while the caret (^) indicates findings specific to females only.

All 999 ICD-9, 3-digit codes plus the mapped BC-specific codes were assessed if there were at least 10 events (for the MS and matched cohort combined) and RRs could be derived from the model. Accordingly the following codes are not shown:5-Other food poisoning (bacterial), 17-Tuberculosis of other organs, 42-Human immunodeficiency virus (HIV) infection with specified conditions, 30- Leprosy, , 162-Malignant neoplasm of trachea, bronchus and lung, 165-Malignant neoplasm of other and ill-defined sites within the respiratory system and intrathoracic organs, 223-Benign neoplasm of kidney and other urinary organs, 240-Simple and unspecified goitre, 241-Nontoxic nodular goitre, 255-Disorders of adrenal glands, 336-Other diseases of spinal cord, 350-Trigeminal nerve disorders, 357-Inflammatory and toxic neuropathy, 369-Blindness and low vision, 424-Other diseases of endocardium, 434-Occlusion of cerebral arteries, 435-Transient cerebral ischaemia, 436-Acute but ill-defined cerebrovascular disease, 453-Other venous embolism and thrombosis, 458-Hypotension, 520- Disorders of tooth development and eruption, 583-Nephritis and nephropathy, not specified as acute or chronic, 586- Renal failure, unspecified, 603-Hydrocele(#), 617- Endometriosis, 659-Other indications for care or intervention related to labour, 660-Obstructed labour, 685-Pilonidal cyst, 799-Other ill-defined and unknown causes of morbidity and mortality, 803-Other and unqualified skull fractures, 805-Fracture of vertebral column without mention of spinal cord lesion, 808-Fracture of pelvis, 913-Superficial injury of elbow, forearm and wrist, 929-Crushing injury of multiple and unspecified sites,993-Effects of air pressure, 996-Complications peculiar to certain specified procedures, and 998-Other complications of procedures, not elsewhere classified.

© 2025 Ruiz-Algueró M et al. *JAMA Network Open*.

eFigure 1. Flow chart summarizing the selection of the multiple sclerosis (MS) cases.

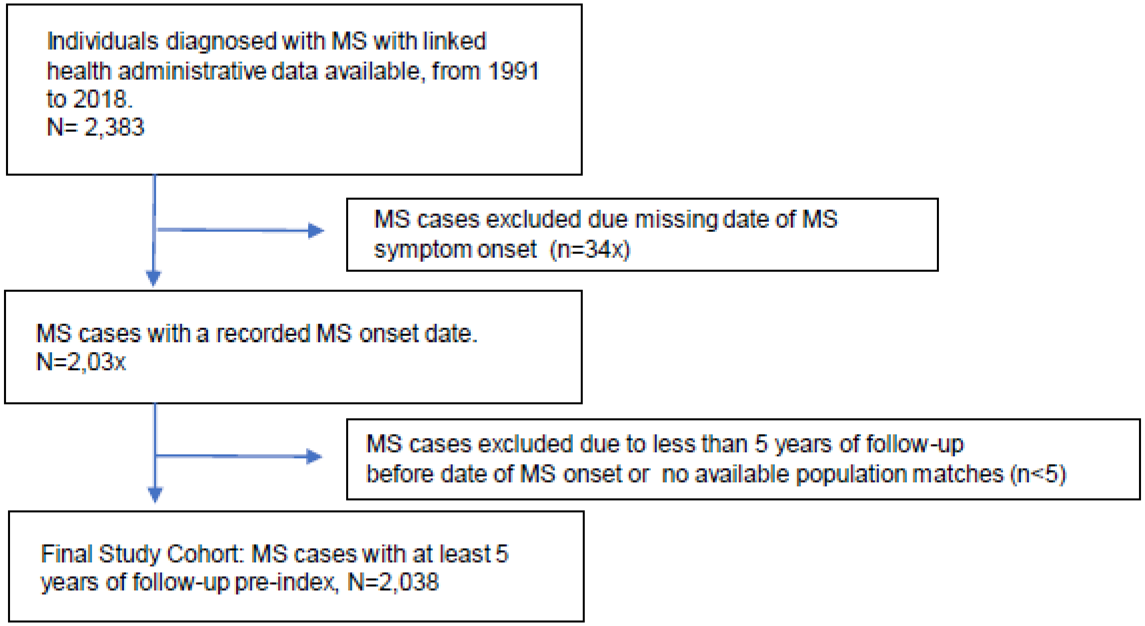

Key: BC, British Columbia. Due to privacy and data access requirements, small cells (<5) cannot be shown. To ensure back-calculation cannot occur 'x' indicates suppression of exact population numbers.
The date of the first MS-specific or demyelinating disease-related International Classification of Diseases code, or disease-modifying drug prescription filled defined the MS onset date.

© 2025 Ruiz-Algueró M et al. *JAMA Network Open*.

eFigure 2. Physician Visits by ICD-9 Chapter in the 15 years preceding multiple sclerosis (MS) symptom onset.

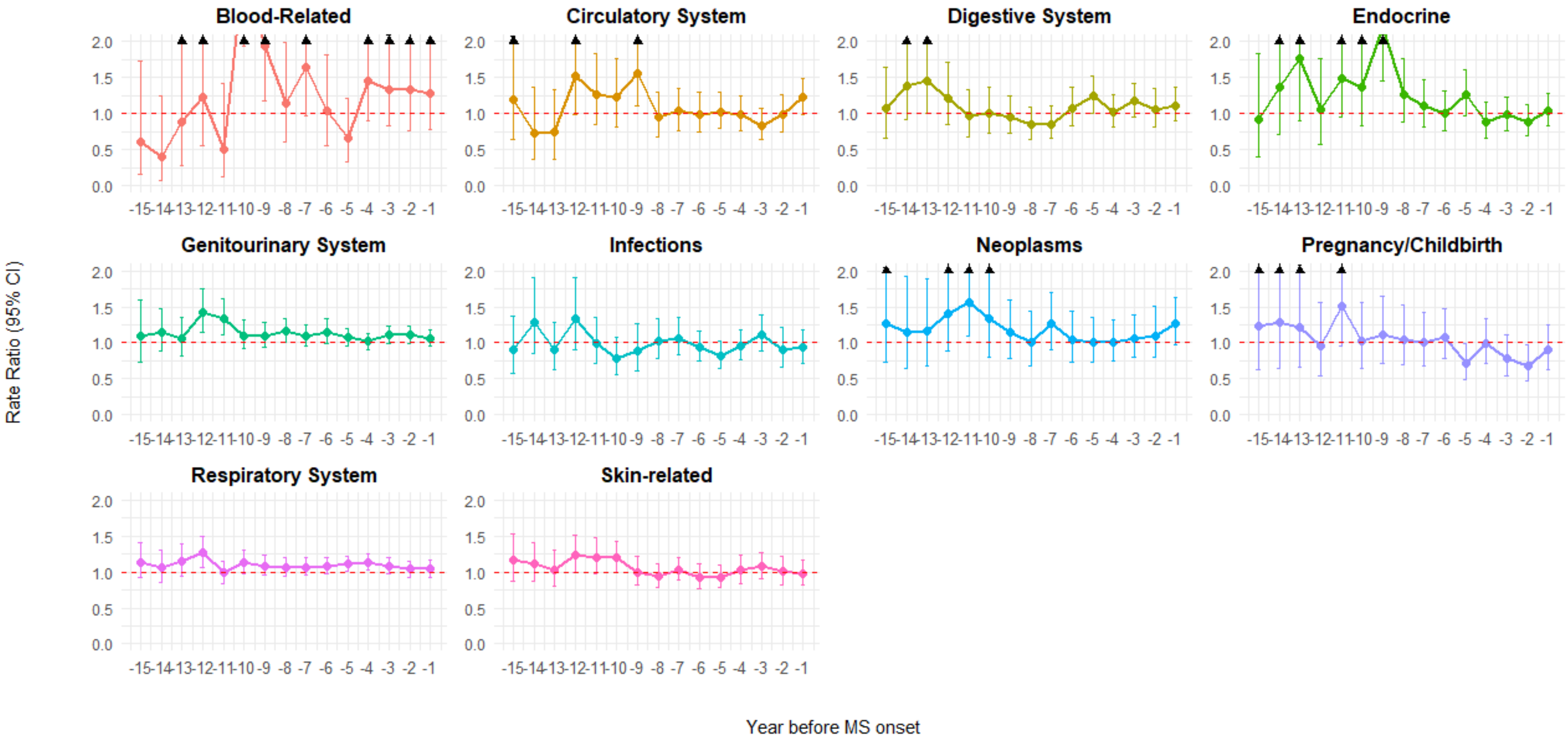

Vertical bars indicate the 95% CI; CI: Confidence Interval. Two ICD-9 chapters (Congenital Anomalies, Chapter 15; and Conditions of the Perinatal Period, Chapter 16) were suppressed as few events occurred and no statistically significant associations across any time point were found. Pregnancy and Childbirth were assessed in females only. Only ICD-9 chapters with more than 10 events (PwMS and the matched cohort without MS combined), across all years in the assessed period, have been plotted. Top black arrows represent upper CI outside the range of the figure.

© 2025 Ruiz-Algueró M et al. *JAMA Network Open*.

eFigure 3. Physician Visits by Specialty in the 15 years preceding multiple sclerosis (MS) symptom onset.

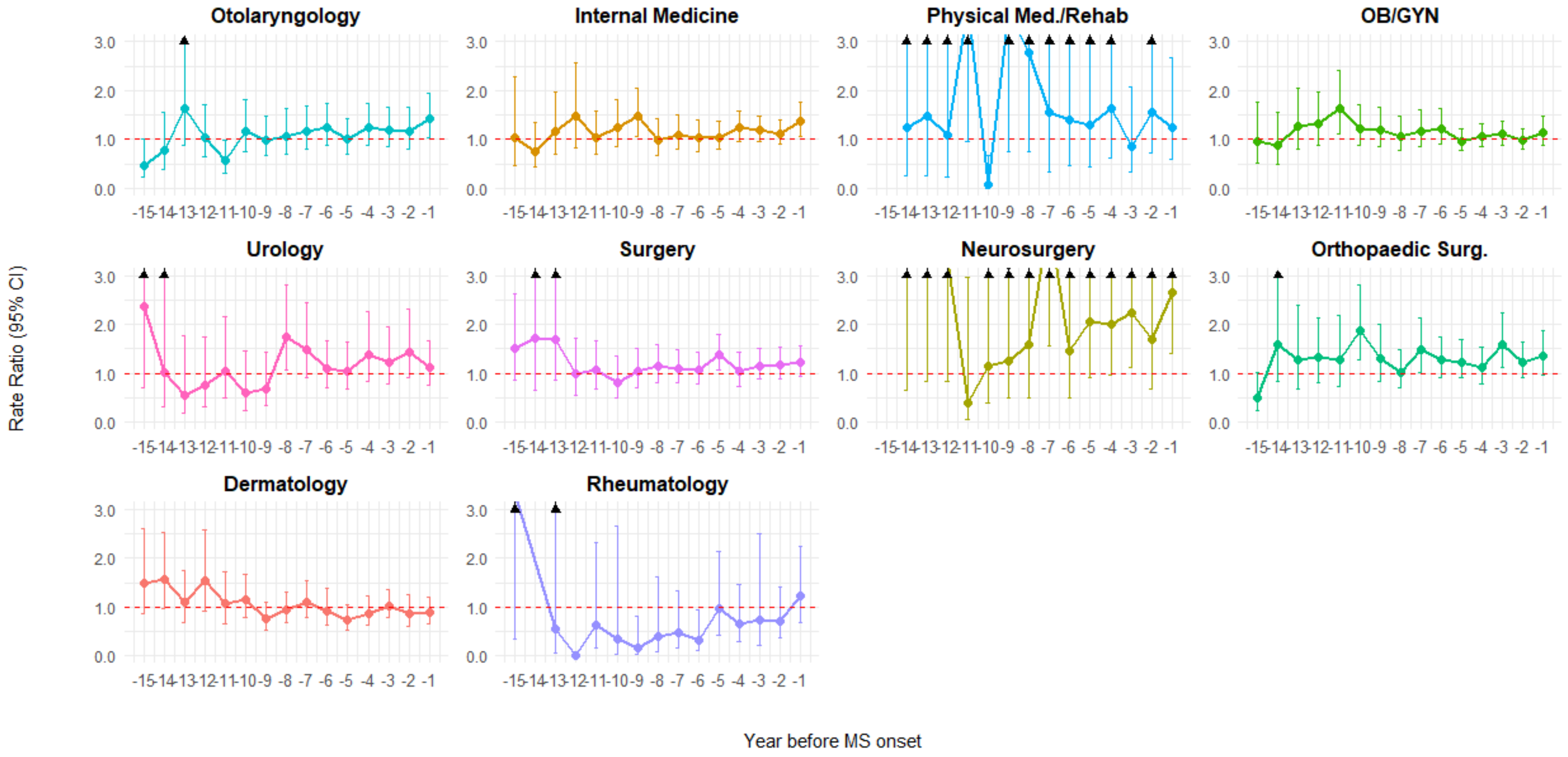

Vertical bars indicate the 95% CI; CI: Confidence Interval. Only specialties with more than 10 events (PwMS and the matched cohort without MS combined), across all years in the assessed period, have been plotted (accordingly, Neuropsychiatry,Cardiology, Endocrinology, Gastroenterology and Nephrology are not shown). OB/GYN assessed in female only. Top black arrows represent upper CI outside the range of the figure. A list of assessed specialties is provided in Supplementary eTable 3.

© 2025 Ruiz-Algueró M et al. *JAMA Network Open*.

eFigure 4. Higher Health Care Use 14 to 15 Years Before Multiple Sclerosis (MS) Symptom Onset: Matched Clinical Cohort Study

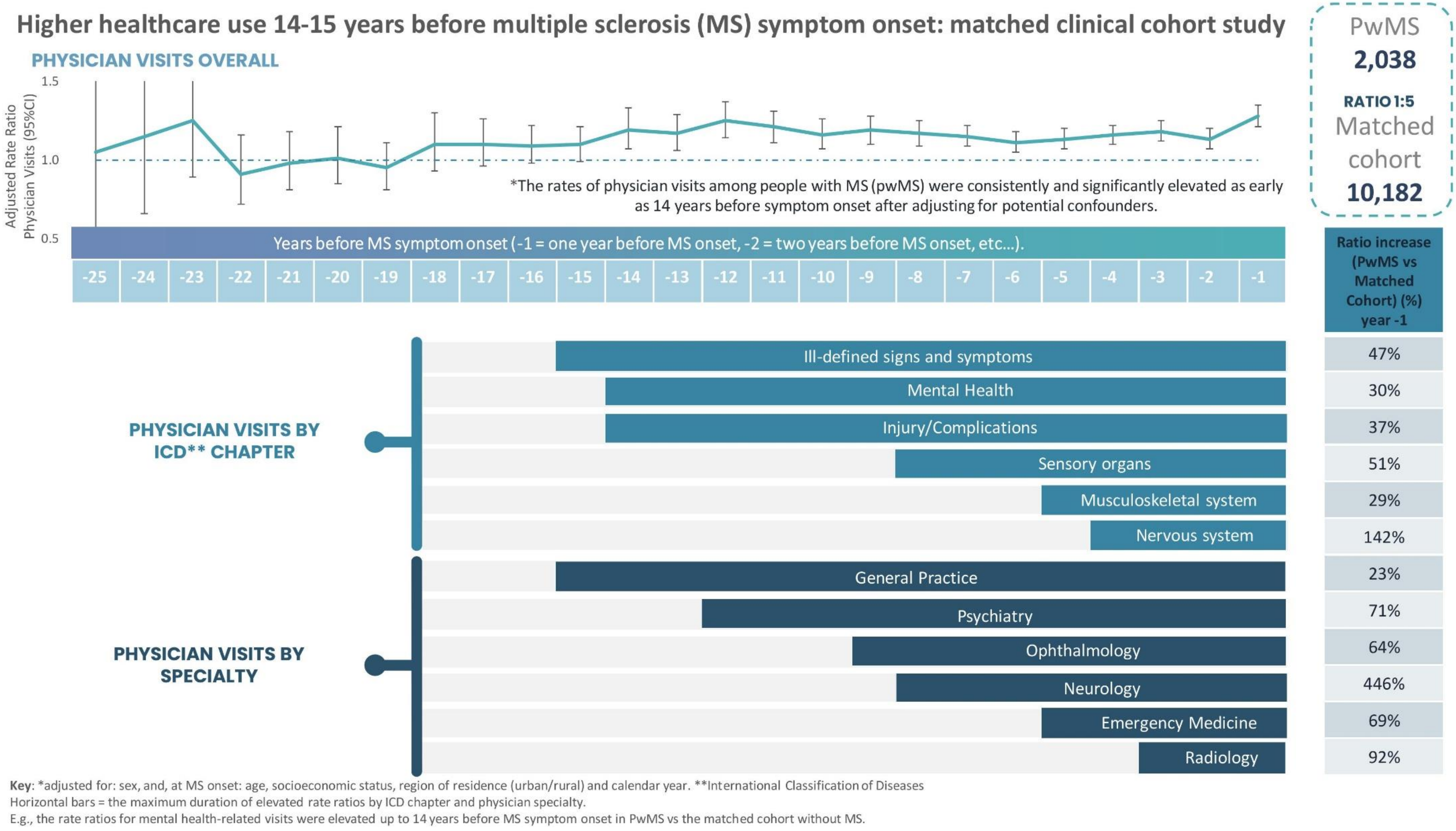

© 2025 Ruiz-Algueró M et al. *JAMA Network Open*.
