# Supplementary material for: Health Care Use Before Multiple Sclerosis Symptom Onset
Source: JAMA Netw Open. 2025 Aug 1;8(8):e2524635. doi: 10.1001/jamanetworkopen.2025.24635 (PMC12317355; doi:10.1001/jamanetworkopen.2025.24635)
Supplement: Supplement 3. — Data Sharing Statement [file jamanetwopen-e2524635-s003.pdf]

Data Sharing Statement

Ruiz-Algueró. Health Care Use Before Multiple Sclerosis Symptom Onset. *JAMA Netw Open*. Published August 01, 2025. doi:10.1001/jamanetworkopen.2025.24635

Data
Data available: No

Additional Information
Explanation for why data not available: DATA SHARING STATEMENT Access to data provided by the data stewards is subject to approval but can be requested for research projects through the data stewards or their designated service providers. As we are not the data custodians, we are not authorized to make these data available. With the appropriate approvals, data may be accessed through Population Data BC.
